# Field data implicating *Culicoides stellifer* and *Culicoides venustus* (Diptera: Ceratopogonidae) as vectors of epizootic hemorrhagic disease virus

**DOI:** 10.1186/s13071-019-3514-8

**Published:** 2019-05-23

**Authors:** Bethany L. McGregor, Kristin E. Sloyer, Katherine A. Sayler, Olivia Goodfriend, Juan M. Campos Krauer, Carolina Acevedo, Xinmi Zhang, Derrick Mathias, Samantha M. Wisely, Nathan D. Burkett-Cadena

**Affiliations:** 1 Florida Medical Entomology Laboratory, University of Florida, Vero Beach, FL USA; 2 Wildlife Ecology and Conservation Department, University of Florida, Gainesville, FL USA; 3 Department of Large Animal Clinical Sciences, University of Florida, Gainesville, FL USA; 4 Department of Entomology and Plant Pathology, Auburn University, Auburn, AL USA; 5 Department of Entomology, University of California Riverside, Riverside, CA USA

**Keywords:** *Culicoides stellifer*, *Culicoides venustus*, hemorrhagic disease, epizootic hemorrhagic disease virus, *Odocoileus virginianus*, white-tailed deer

## Abstract

**Background:**

Epizootic hemorrhagic disease virus (EHDV) is an *Orbivirus* of veterinary importance which is transmitted by biting midges of the genus *Culicoides* (Diptera: Ceratopogonidae) to ruminants. *Culicoides sonorensis* Wirth & Jones, the only confirmed vector of EHDV in the USA, is rare in the southeastern states where transmission persists, suggesting that other *Culicoides* species transmit EHDV in this region. The present study aimed to determine which *Culicoides* species transmitted EHDV in Florida and Alabama, two states in the southeastern USA. Viral RNA was detected in field-collected midges using molecular methods. These data are presented alongside data on *Culicoides* blood meal analysis, white-tailed deer (*Odocoileus virginianus*) aspiration, and seasonality to demonstrate an interaction between potential vector species and EHDV hosts.

**Results:**

Out of 661 pools tested, 20 pools were positive for EHDV viral RNA, including six pools from *Culicoides stellifer* (Coquillett) and 14 pools from *Culicoides venustus* Hoffman. The overall infection rate was 0.06% for *C. stellifer* and 2.18% for *C. venustus*. No positive pools were identified for a further 17 species. Serotypes identified in *Culicoides* included EHDV-2, EHDV-6, and coinfections of EHDV-2 and EHDV-6 and were identified in similar proportions to serotypes in deer at 3 of 4 deer farms. Viral detections conducted in Alabama also identified one positive pool of *C. venustus*. Blood meal analysis revealed that both *Culicoides* species fed on white-tailed deer (verified through aspiration), fallow deer, and elk, species for which EHDV viremia has been documented. Seasonality data indicated that both species were present throughout the period in which viral transmission occurred to EHDV hosts in 2016 in addition to the 2017 epizootic.

**Conclusions:**

Our finding of EHDV positive pools of field-collected *C. stellifer* and *C. venustus* and an interaction between these species and EHDV hosts satisfy two of the four criteria for vector incrimination as set by the World Health Organization. Determining the vectors of EHDV is an important step towards developing sound strategies for the control of vector *Culicoides* and management of EHDV in the southeastern USA.

## Introduction

Epizootic hemorrhagic disease virus (EHDV) is an *Orbivirus* of veterinary importance which occurs worldwide in susceptible hosts where competent *Culicoides* Latreille (Diptera: Ceratopogonidae) vectors exist, including Africa, Asia, Australia, Europe, North America, and South America [1–4]. The primary mammalian host species affected by the virus are wild ungulates, while domestic ruminants such as cattle do not typically succumb to disease [1]. Certain serotypes and strains of this pathogen, such as the Ibaraki strain of EHDV-2, have greater pathogenicity to cattle, although outbreaks associated with this strain have been isolated [5]. This is in direct contrast to the closely related bluetongue virus (BTV), which causes considerable morbidity and mortality in domestic sheep, abortions in cows, and decreased milk production in dairy cattle [6–10]. Due to the greater economic impact of BTV, extensive research has been conducted on the pathogen and arthropod vector species for this disease system. However, EHDV has not received the same amount of research due to its envisaged low impact on economically valuable industries. Important questions remain regarding the ecology and epidemiology of EHDV in North America, including which *Culicoides* species are transmitting this pathogen in regions of the USA where documented vectors are absent.

Deer farming is a growing industry that is being impacted by EHDV in the USA. While deer have historically been used for meat and musk production in New Zealand and China respectively, the industry has been slow to develop in other countries [11–13]. In the USA, deer farming is a young but growing industry. Estimates place the economic impact of deer farming in the USA at $7.9 billion annually, supporting greater than 56,000 jobs [14]. EHDV often results in mortality of wild and farmed deer worldwide, with farmers occasionally losing up to 80% of their herd [15]. Although autogenous vaccines are available for use within adjacent herds, evidence indicates that commercially available EHDV vaccines do not produce a sufficient humoral response for protection [16], resulting in economic losses due to the purchase of ineffective vaccines and loss of valuable animals. For this reason, the need to better understand EHDV epidemiology in the USA has been prioritized [17].

EHDV is transmitted by small (1–3 mm) hematophagous flies in the genus *Culicoides*, with only one confirmed vector species in the USA, *Culicoides sonorensis* Wirth & Jones [18, 19]. This species is common and abundant throughout the western and midwestern states west of the Missouri river [20]. However, in the southeastern states, where EHDV persists annually, *C. sonorensis* is rare, as evidenced by the lack of this species in multiple large-scale *Culicoides* surveys [21, 22]. The low abundance of *C. sonorensis* indicates that alternative vector species are likely present in this region of the USA where EHDV cases have been documented. A few species have been implicated as potential vector species of EHDV in the southeastern states based on their abundance and presence near affected host species. These species include *Culicoides debilipalpis* Lutz, *Culicoides obsoletus* Meigen, *Culicoides paraensis* Goeldi, *Culicoides spinosus* Root & Hoffman and *Culicoides stellifer* (Coquillett) [21–23]. While the abundance of these species is an important consideration when incriminating potential vectors, this factor alone cannot be used to implicate a species as a vector of EHDV. Other criteria that should be fulfilled to confirm a putative vector species include (i) recovering virus from field-collected individuals without visible blood in the gut; (ii) demonstrating that the arthropod can become biologically infected after an infected blood meal; (iii) demonstrating the arthropod’s ability to transmit the virus; and (iv) showing a significant association between the implicated arthropod and the affected host population [24]. EHDV transmission studies in *Culicoides* are lacking, due primarily to the difficulties in colonizing *Culicoides* species and inducing blood-feeding in the laboratory [25, 26]. Despite this lack of information, the impact of hemorrhagic disease (HD) on deer farmers necessitates the identification of potential vectors so that management plans can be developed and implemented.

Due to the challenges associated with conducting laboratory vector competence studies on *Culicoides*, field-based evidence is vitally important to incriminating vectors in a diverse community (> 50 *Culicoides* spp. are present in Florida). This study was aimed at investigating the potential vector(s) of EHDV among white-tailed deer (*Odocoileus virginianus*) in the southeastern USA using available vector incrimination criteria. Detection of EHDV RNA by qRT-PCR from field-collected biting midges and deer showing signs of disease during an epizootic in northern Florida and from field-collected midges in Alabama during a non-outbreak period was performed to quantify field infection rates. Examining data from blood meal analysis, live animal aspiration, and midge seasonal abundance collected prior to an epizootic permitted inference on host association of implicated *Culicoides* species. Over the long term, once vector species have been identified, we can begin to fill gaps in knowledge on their ecology enabling the development of more targeted approaches to biting midge control.

## Methods

### *Culicoides* sampling and virus detection during EHDV epizootic in northern Florida

*Culicoides* midges and animal tissues were collected from five deer farms with suspected EHDV cases based on clinical presentation in affected animals in northern Florida from August-October in 2017 (Table 1). Coordinates to the specific farm sites are not provided per the wishes of the private landowners whose properties were sampled. Coordinates to the nearest county center were 30.7151°N, 85.1894°W (Jackson), 30.1508°N, 84.8568°W (Liberty), 30.5563°N, 84.6479°W (Gadsden, two farms), and 30.4312°N, 83.8897°W (Jefferson). Insects were sampled overnight using CDC miniature light traps (Model 2836BQ, BioQuip Inc., Rancho Dominguez, USA) baited with either incandescent yellow light bulbs or LED black light arrays (model 2790V390, BioQuip Inc., Rancho Dominguez, USA) and carbon dioxide (solid dry ice). Four traps were used per night at the Jackson, Liberty, Gadsden-2, and Jefferson county farms and five traps were used at the Gadsden-1 site. All collections were made in response to reports of EHDV-related deer mortality beginning in September, except those at the Gadsden-1 site where trapping was ongoing and predated the first EHDV-related deer death. *Culicoides* were collected into ethanol and transported on dry ice back to the Florida Medical Entomology Laboratory for processing. *Culicoides* were identified based upon external morphology of the female [27], then pooled by species, location, and date (maximum pool size of 50 individuals). Midges were also pooled by parity, with pools containing only parous and gravid females [28]. Due to difficulties in identifying parity in *Culicoides venustus* Hoffman, nulliparous females were included in pools. No visible blood was present in any of the midges tested for virus. Samples were stored in ethanol and identified on dry ice prior to being pooled and transferred to 2 ml microcentrifuge tubes containing HyClone medium 199 with Earle’s balanced salt solution (GE Healthcare Life Sciences, Chicago, IL, USA) for homogenization. Samples were homogenized using a TissueLyser (Qiagen, Valencia, CA, USA) set at 19.5 Hz for three minutes or using a Bullet Blender Storm 24 (Next Advance, Troy, NY, USA) at speed four for three min. Viral RNA was extracted from lysate using the QiaAmp Viral RNA Mini kit following kit protocols (Qiagen, Valencia, CA, USA).

**Table 1:** Summary of sampling locations, sampling frequency, and EHDV-positive samples pulled from dead deer in 2017

County	Month	Days sampled	Trap nights	Deer sampled
Jackson	September	2	8	2
	October	2	8	0
Liberty	September	0	0	13
	October	6	20	2
Gadsden-1	August	3	12	0
	September	9	45	1
	October	8	40	5
Gadsden-2	September	3	12	1
	October	3	12	0
Jefferson	September	3	12	4
	October	5	20	1
Total		44	189	29

*Notes*: Samples were taken from four counties in Florida: Jackson, Liberty, Gadsden and Jefferson. Trapping was conducted using CDC miniature light traps baited with either UV or incandescent light and, when available, CO_2_. Deer EHDV positives were first detected by gross pathology of the animal and tissues followed by RT-PCR confirmation of EHDV viral RNA. Trapping at Jackson, Liberty, Gadsden-2 and Jefferson took place only after an initial EHDV-related mortality from September-October. August data is included for Gadsden-1 due to ongoing trapping at this location at the time of the EHDV outbreak

Viral RNA was amplified using a multiplex qRT-PCR protocol for BTV and EHDV using the SuperScript III Platinum One-step qRT-PCR kit (Thermo Fisher Scientific, Waltham, MA, USA). Reagents per sample included 2.2 µl molecular grade water, 12.6 µl 2× reaction mix, 1 µl each 10 µM BTV forward and reverse primer, 0.8 µl each 20 µM EHDV forward and reverse primer, 0.4 µl each of 10 µM FAM-labelled BTV probe and 20 µM Texas Red labelled EHDV probe, 0.8 µl Platinum *Taq*/SuperScript III reverse transcriptase mix, and 5 µl RNA template (50–100 ng/µl concentration). BTV and EHDV primer and probe sequences were from Wernike et al. [29]. Cycling conditions were modified from Wernike et al. as follows: reverse transcription at 48 °C for 10 min, initial denaturation for 10 min at 95 °C, followed by 40 cycles of 95 °C for 15 s, 57 °C for 45 s, and 68 °C for 45 s. All reaction plates contained EHDV positive control RNA from the Centers for Disease Control and Prevention and a molecular biology grade water negative control. The serotype of EHDV vRNA positive samples was identified in a subsequent reaction using primers and probes for EHDV-1, EHDV-2, and EHDV-6 described by Maan et al. [30] The 25 µl assay was modified from the previously published method as follows: 2× Vet-MAX-Plus One-Step qRT-PCR (Thermo Fisher Scientific, Waltham, MA, USA) was added to the reaction mixture and 5 µl of RNA was utilized as template. Amplification was carried out using an ABI 7500 FAST system (Applied Biosystems, Waltham, MA, USA) using slightly modified conditions as follows: 48 °C for 10 min reverse transcription, followed by 95 °C for 10 min, and 45 cycles of 95 °C for 15 s and 60 °C for 60 s.

### White-tailed deer sampling and virus detection during the 2017 epizootic

Field necropsies were performed on farmed white-tailed deer that succumbed to disease following clinical signs of HD infection (edema, inappetence and lethargy) at the same five farms where *Culicoides* sampling was conducted (Table 1). Whole blood was collected and transferred in RNase-free sterile tubes to the University of Florida Cervidae Health Research Initiative (CHeRI) for further analysis.

RNA was extracted from whole blood using the QIAamp Viral RNA mini kit (Qiagen, Valencia, CA, USA) according to the manufacturer’s instructions. Cycling conditions, primers, and probes for multiplex qRT-PCR detection of EHDV and BTV were the same as for the *Culicoides* viral RNA detection described above. The master mix reagents were adjusted for use with the VetMax-Plus Multiplex One-Step RT-PCR kit (Thermo Fisher Scientific, Waltham, MA, USA) and VetMAX Xeno RNA internal control (Thermo Fisher Scientific, Waltham, MA, USA) as follows: 12.5 µl 2× multiplex RT-PCR buffer, 1 µl each of 10 µM BTV forward and reverse primer, 0.2 µl BTV FAM labelled probe, 0.75 µl each of 20 µM EHDV forward and reverse primer, 0.125 µl EHDV Texas Red labelled probe, 1 µl Xeno RNA internal control, 0.375 µl molecular grade water, and 2.5 µl 10× multiplex RT-PCR enzyme. All reaction plates included positive control RNA for EHDV-1, EHDV-2, and EHDV-6 provided by the Southeastern Cooperative Wildlife Disease Study, a molecular biology grade water negative control, and a non-template Xeno internal control. PCR protocols for serotyping white-tailed deer blood samples were identical to the protocols described above for *Culicoides* pools.

### Statistical analysis of virus data

Maximum likelihood estimates (MLE) were calculated to determine the infection rate and 95% confidence intervals at each site where positives were recovered [31]. This metric estimates infection rates based on probabilistic models following a binomial distribution and can be adapted for use with variable pool sizes [32–34]. A Fisher’s exact test was run to analyze whether serotype was associated with midge species tested. Fisher’s exact tests were also used to compare the distribution of serotypes in deer with serotypes in midge pools at each farm. In order to determine what variables might predict recovery of virus positive pools, negative binomial regression models were fitted, and the best model was selected by Akaike information criterion (AIC) utilizing a backwards stepwise selection method. Variables included the day of the year, site, light type, presence of CO_2_, abundance of *C. stellifer*, abundance of *C. venustus*, and total abundance of other species collected. The response variable was the total number of positive EHDV pools. Light type and CO_2_ use were not recorded for Gadsden-2 and Jefferson sites from September 18–20, resulting in the removal of these dates from this analysis. All analyses were run using R studio (The R Foundation for Statistical Computing, version 3.3.3).

### Pre-outbreak field studies of host use and *Culicoides* seasonal abundance

In order to identify host-use patterns, midges were aspirated at the Gadsden-1 site from tame white-tailed deer in pens from June 2015 through September 2016. Collections were made at three time points weekly (dawn, midday, dusk) and were conducted on any approachable animals for 10 minutes per session. The aspirator was swept over the entire body during this period. Aspirator design was an acrylic tube with a computer fan powered by a 12V battery. Collections were made directly into a plastic collection cup with a wire mesh bottom and stored at −20 °C until analyzed. All midges collected were identified to species using morphological keys [27].

### Blood meal analysis

Midges were collected twice weekly at the Gadsden-1 site between July 2015 and August 2017 to quantify seasonal abundance patterns and host use. This site is largely composed of a 200-ha hunting preserve harboring a variety of Bovidae and Cervidae species, with two areas of penned white-tailed deer present. Midges were collected at 20 sites throughout the preserve using CDC miniature light traps with LED black light arrays set 1.62 m above the ground. Between November-March, trapping was restricted to 10 sites twice per week. At 10 trap sites, additional elevated trapping at 6 m in 2016 and 9 m in 2017 was conducted using traps suspended from trees [35].

All blood-engorged midges collected were analyzed to determine blood meal origin down to the level of vertebrate species using published protocols [36]. In addition to looking at blood meals on the primary EHDV host, white-tailed deer, the blood meal results for elk (*Cervus* spp.) and fallow deer (*Dama dama*) were also compiled. Studies have indicated that both elk and fallow deer can be viremic carriers of EHDV [37, 38].

### Seasonal abundance of *C. stellifer* and *C. venustus*

During 2016, animal mortality data and midge abundance data were recorded from Gadsden-1 from January-December. Collections were made twice weekly using the same 20 CDC miniature light traps at the Gadsden-1 site that were used for blood-engorged *Culicoides* collection (reduced to 10 traps during the months of November-March). Total collections of female *C. venustus* and *C. stellifer* from 2016 were compiled temporally to determine periods of high midge activity. Data on EHDV-related deer mortality at this farm were also compiled from 2016.

### *Culicoides* sampling and EHDV detection in biting midges from Alabama

*Culicoides* sampling was conducted from late June through mid-November, 2016, at nine sites within the Piedmont Research Unit of the Alabama Agricultural Experiment Station in eastern central Alabama (Site 1: 32.8257°N, 85.649°W; Site 2: 32.8256°N, 85.6498°W; Site 3: 32.8249°N, 85.6504°W; Site 4: 32.8252°N, 85.6464°W; Site 5: 32.8256°N, 85.6461°W; Site 6: 32.8262°N, 85.6458°W; Site 7: 32.8217°N, 85.6497°W; Site 8: 32.8212°N, 85.6497°W; Site 9: 32.8212°N, 85.6492°W). Six of the nine sites were located within a 430-acre enclosure housing a captive white-tailed deer population. At each site a CDC miniature light trap baited with CO_2_ was set every other week from dusk until dawn for 23 weeks and traps with three different light sources (incandescent bulb, UV black light fluorescent tube, and UV LED array) were rotated among sites. *Culicoides* from all traps were transported to Auburn University in a cooler with ice packs, sorted and identified to species on ice using external morphology of the female [27], pooled by species, and divided into male and female groups. Biting midges from UV LED traps were prioritized for EHDV screening and were therefore stored at −80 °C in RNAlater (Thermo Fisher Scientific, Waltham, MA, USA). Females from these traps were screened for presence of EHDV using the following protocol. *S*amples with fewer than 15 individuals of a specific species, trap date, and sampling site were kept together for extraction as a single pool, while ones containing more than 15 individuals were divided into multiple pools of *n* ≤ 15. Immediately prior to RNA extraction, RNALater was removed from each pool and midges were homogenized in RiboZol RNA extraction reagent (VWR International, Radnor, PA, USA). RNA extractions proceeded following the manufacturer’s protocol for RNA isolation. To remove genomic DNA contamination, RNA extractions were treated with DNase I, followed by RNA cleanup with the RNeasy Mini Kit (Qiagen) following the manufacturer’s protocol for DNase digestion in solution. Pools were screened for EHDV as described above with a few modifications. Instead of using a one-step protocol, cDNA was synthesized from total RNA using random hexamers and the RevertAid First-Strand cDNA Synthesis kit (Thermo Fisher Scientific, Waltham, MA, USA). Resulting products were used as template for qRT-PCR with the EHDV primers and probe described by Wernike et al. [29]. Each reaction included 2.6 µl molecular grade water, 10.0 µl 2× iQ Multiplex Powermix (Bio-Rad, Hercules, CA, USA), 1.0 µl each 10 µM EHDV forward and reverse primer, 0.4 µl 20 µM EHDV probe, and 5 µl cDNA template. Cycling conditions included an initial denaturation of 95 °C for 3 min, followed by 45 cycles of 95 °C for 10 s and 57 °C for 1 min on a Bio-Rad CFX96 touch real-time PCR detection system.

## Results

### Virus detection in field-collected midges during an EHDV epizootic

**Fig. 1:**
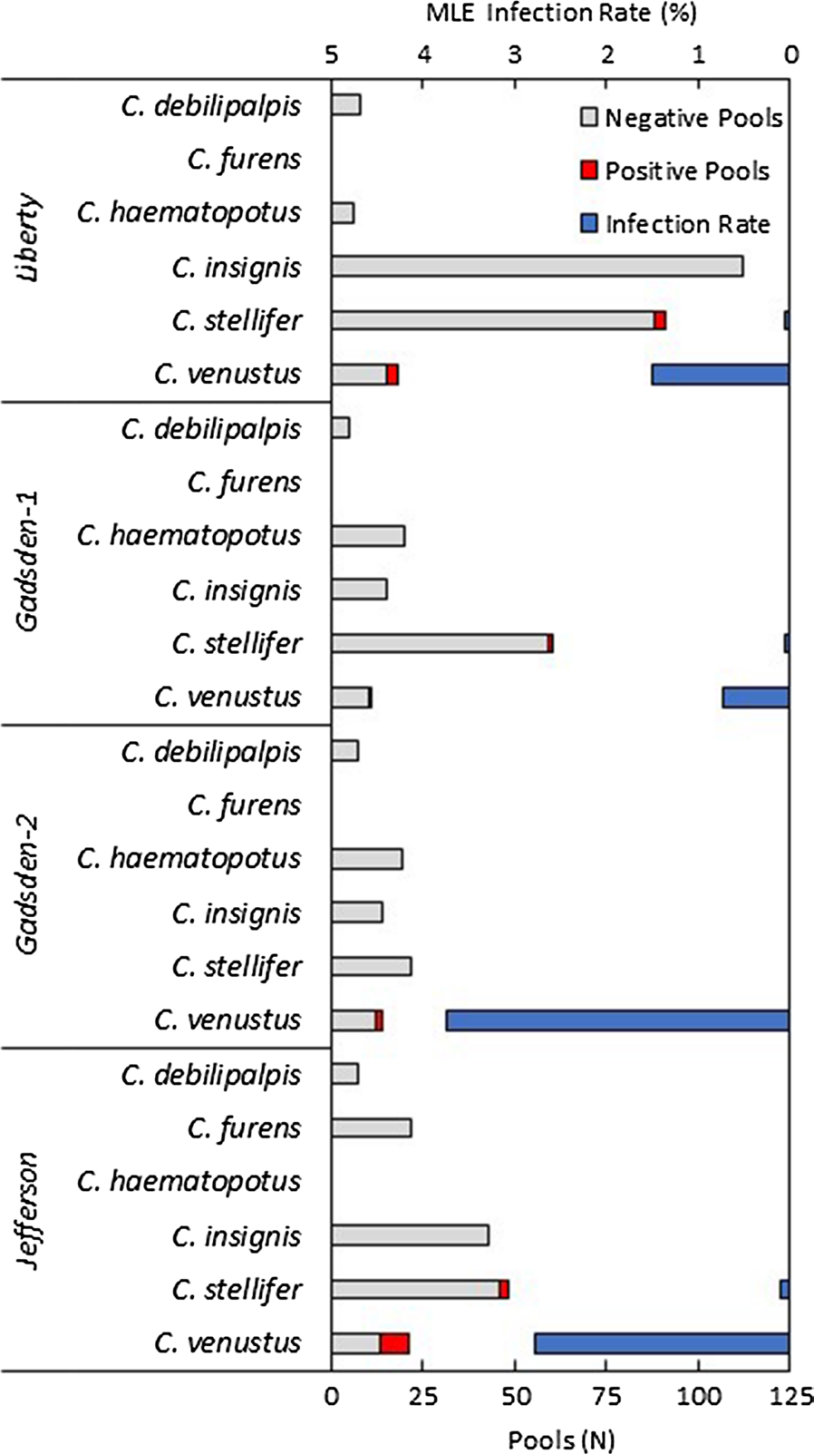
EHDV infection rate of *Culicoides* at four Florida deer farms during HD epizootic, 2017. Midges were collected using CDC miniature light traps. Nineteen species were tested for EHDV viral RNA by qRT-PCR, six of the most abundant species are shown. Infection rates were calculated using maximum likelihood estimates (MLE) adjusted for use with variable pool sizes

From September-October, 16 traps were set at Jackson, 20 at Jefferson, 24 at Gadsden-2, and 32 at Liberty. Trapping took place from August-October at Gadsden-1 with 97 total trap nights during this time. In total, 19,000 biting midges (661 pools) from 19 total species were screened for the presence of EHDV RNA from five deer farms during the course of an HD outbreak in northern Florida, resulting in 20 EHDV-positive pools (Table 2). All positive pools were from two species, *C. venustus* (14 positive pools, 70%) and *C. stellifer* (6 positive pools, 30%) identified from four of the farms, including the farms in Liberty and Jefferson Counties and both farms in Gadsden County (Fig. 1). The farm in Liberty had six total positive pools out of 265 pools tested, three of the positives were from pools of *C. stellifer* (MLE (maximum likelihood estimate) infection rate = 0.05%, 95% CI: 0.01–0.17%) and three were from pools of *C. venustus* (MLE = 1.50%, 95% CI: 0.41–4.08%). Gadsden-1 had two positive pools, one *C. stellifer* (MLE = 0.05%, 95% CI: 0.00–0.23%) and one *C. venustus* (MLE = 0.73%, 95% CI: 0.04–3.70%), out of 113 tested. Gadsden-2 had two positive pools, both *C. venustus* (MLE = 3.75%, 95% CI: 0.67–12.61%), out of 79 pools tested. Jefferson had the most positives with ten positive pools, two from *C. stellifer* (MLE = 0.10%, 95% CI: 0.02–0.33%) and eight from *C. venustus* (MLE = 2.78%, 95% CI: 1.42–5.29%), out of 176 tested. No other *Culicoides* species were positive for EHDV vRNA (Fig. 1). Most positive pools were identified as EHDV serotype-6 (*n* = 12, 60%; Table 3). Six of the pools were identified as EHDV serotype-2 (30%). Two pools, both of *C. venustus* collected in Jefferson County, were typed to both EHDV serotype-6 and serotype-2. None of the samples were identified as the other known EHDV serotype present in Florida, EHDV-1. Serotype was independent of midge species tested (Fisher’s exact test, *P* = 0.351). *Culicoides venustus* and *C. stellifer* were infected with each serotype in similar proportions.

**Table 2:** Total *Culicoides* sampled, number of pools per species and EHDV positive pools during the EHDV epizootic in northern Florida from August-October, 2017

**Species**	Jackson	Liberty	Gadsden-1	Gadsden-2	Jefferson
*C. arboricola*	0	3 (0/3)	1 (0/1)	1(0/1)	24 (0/6)
*C. baueri*	0	2 (0/2)	0	0	40 (0/4)
*C. bickleyi*	0	0	0	0	11 (0/2)
*C. biguttatus*	1 (0/1)	240 (0/11)	0	0	0
*C. crepuscularis*	2 (0/2)	0	0	0	0
*C. debilipalpis*	1 (0/1)	38 (0/8)	13 (0/5)	23 (0/7)	33 (0/7)
*C. furens*	0	0	0	0	648 (0/22)
*C. haematopotus*	5 (0/1)	10 (0/6)	426 (0/20)	480 (0/19)	0
*C. insignis*	131 (0/8)	4790 (0/112)	142 (0/15)	125 (0/14)	1528 (0/43)
*C. mississippiensis*	0	0	0	0	175 (0/11)
*C. nanus*	0	0	0	2 (0/1)	0
*C. pallidicornis*	0	0	0	0	22 (0/3)
*C. pusillus*	0	0	0	2 (0/1)	1 (0/1)
*C. scanloni*	0	0	2 (0/1)	0	0
*C. spinosus*	2 (0/1)	260 (0/13)	0	0	54 (0/8)
*C. stellifer*	116 (0/9)	4016 (3/91)	2065 (1/60)	658 (0/22)	2032 (2/48)
*C. torreyae*	1 (0/1)	0	0	0	0
*C. variipennis*	0	1 (0/1)	0	0	0
*C. venustus*	4 (0/4)	215 (3/18)	140 (1/11)	60 (2/14)	454 (8/21)
Total	263 (28)	9575 (265)	2789 (113)	1351 (79)	5022 (176)

*Notes*: Each cell contains the total *Culicoides* per species sampled with the number of positive pools over the total pools per site in parenthesis. *Culicoides* were sampled using CDC miniature light traps at five deer farms in four Florida counties: Jackson, Liberty, Gadsden and Jefferson

**Table 3:** Serotypes of EHDV recovered from *C. stellifer*, *C. venustus* and *O. virginianus* (white-tailed deer) during the 2017 outbreak

Serotype	Species	Jackson	Liberty	Gadsden-1	Gadsden-2	Jefferson
2	*C. stellifer*	0	1	0	0	0
	*C. venustus*	0	1	1	1	2
	*O. virginianus*	0	1	2	1	5
6	*C. stellifer*	0	2	1	0	2
	*C. venustus*	0	2	0	1	4
	*O. virginianus*	2	13	5	0	0
2 and 6	*C. stellifer*	0	0	0	0	0
	*C. venustus*	0	0	0	0	2
	*O. virginianus*	0	1	0	0	0

*Notes*: EHDV serotypes 2 and 6 were identified on multiple properties, with some pools containing both serotypes. Pooled midges were collected using CDC miniature light traps during an EHDV outbreak in Northern Florida from August-October, 2017. White-tailed deer samples were taken from individuals that died from EHDV infection

In a negative binomial regression model investigating the effect of site, day of year, light type, presence of CO_2_, *C. stellifer* abundance, *C. venustus* abundance, and total other species abundance on the likelihood of getting a positive midge pool, the model of *C. stellifer* abundance, *C. venustus* abundance, and presence of CO_2_ was the most parsimonious with an AIC of 56.731. The next best models for predicting the likelihood of recovering a virus positive pool were *C. venustus* abundance and CO_2_ presence (AIC = 56.83) and *C. venustus* abundance alone (AIC = 57.224). In all models, *C. venustus* abundance was the single significant factor at *P* < 0.05.

In addition to the EHDV positive pools, one midge pool was positive for the presence of BTV RNA in the multiplex assay. The positive came from a pool of 42 *C. stellifer* collected at Gadsden-2 on September 20, 2017. BTV serotypes were not determined for this isolate. BTV was detected in three deer from Gadsden-1, seven deer from Gadsden-2, and one deer from Liberty during the *Culicoides* sampling period.

### Animal mortality due to EHDV

From early-September to mid-October 2017, a total of 30 HD cases attributable to EHDV serotypes 2, 6 or coinfections with both serotypes were observed at all five deer farms (Table 3). Gross pathology observed in most cases (23/30) included generalized edema and hemorrhages involving different tissues and organs, most frequently being lung, heart, spleen and kidney. Similarly, hemorrhages were observed on the serosal surfaces of the stomach and there were multiple appreciable hemorrhages and intravascular coagulation on the pulmonary arteries. HD was determined to be the cause of death in all cases based on a combination of clinical signs, including the peracute or acute nature of disease, gross pathology and molecular data (all cases were confirmed by detection of viral RNA in the whole blood of suspect animals). At Liberty, 15 cases were confirmed: all were caused by EHDV-6 except a single case of EHDV-2 and a single case of mixed infection (confirmed in multiple tissues) with types 6 and 2. At Gadsden-1, seven deaths caused by infection with EHDV were confirmed: two were caused by EHDV-2 infection and the remainder, all which impacted fawns, were caused by EHDV-6. At Gadsden-2, one death caused by EHDV-2 was confirmed. At Jefferson, five deaths caused by EHDV-2 were confirmed. Four of five individuals were fawns born in 2017. At Jackson, where no positive midge pools were detected, there were two deer deaths caused by EHDV-6.

At Liberty, Gadsden 1, and Gadsden 2 the serotypes identified in deer and midge samples (with *C. stellifer* and *C. venustus* positives combined) were not significantly different (Fisher’s exact test, *P* = 0.292, 1, and 1 respectively). This indicates that the midges were positive for serotypes in similar proportions to the EHDV positive white-tailed deer on these farms. Fisher’s exact test results for the Jefferson farm were significant indicating that the proportion of detected serotypes in midge pools was independent of the serotype composition detected from deer at this farm (*P* *=* 0.026).

### Aspiration

**Fig. 2:**
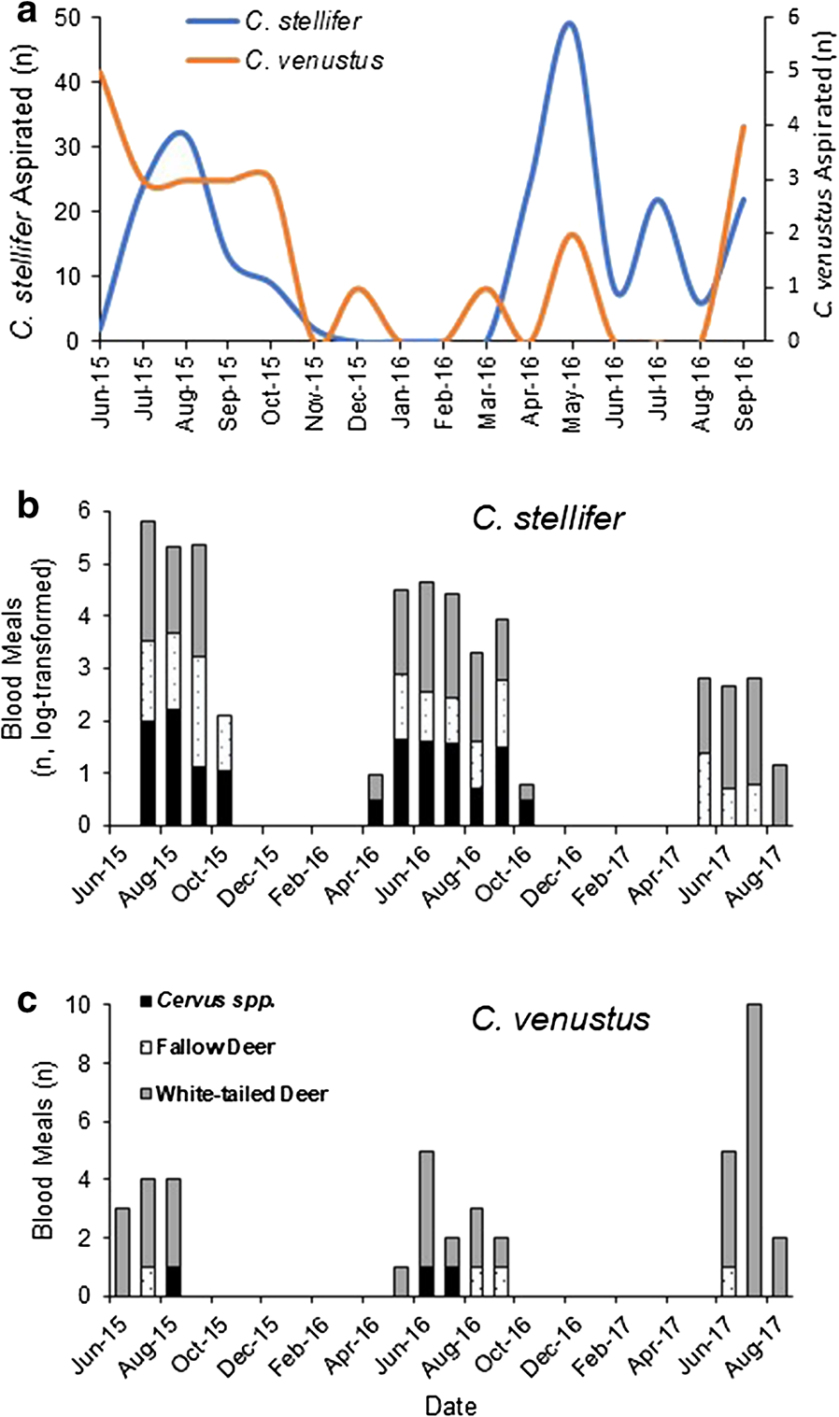
Host use of *Culicoides stellifer* and *Culicoides venustus* at the Gadsden-1 site. Host use was determined by direct aspiration from tame white-tailed deer from June 2015-September 2016 (**a**) and PCR-based blood meal analysis from *C. stellifer* (**b**) and *C. venustus* (**c**) collected from CDC miniature light traps from June 2015-August 2017 at a big game preserve located in Gadsden County, Florida

A total of 685 individuals were collected during live-animal aspirations. Both *C. stellifer* and *C. venustus* were collected from white-tailed deer using live animal aspiration. A total of 25 *C. venustus* and 213 *C. stellifer* were collected from June 2015 through September 2016 (Fig. 2a). In 2015, *C. stellifer* and *C. venustus* were both collected feeding on deer, starting when aspirations began in June and ending in November for *C. stellifer* (*n* = 82 individuals), and December for *C. venustus* (*n* = 18). In 2016, *C. stellifer* was collected feeding on deer from April through September (*n* = 131); *Culicoides venustus* was collected rarely (*n* = 7) compared with *C. stellifer*, with the greatest abundance collected during September (*n* = 4). Other species in aspiration collections included *C. biguttatus* (*n* = 15) and *C. pallidicornis* (*n* = 373), both of which were only documented approaching deer from March-May. *Culicoides debilipalpis* were collected from June through September (*n* = 56) with the greatest collection in August (*n* = 40, 71.4% of total collected). A few individuals of *C. insignis* (*n* = 2) and *C. paraensis* (*n* = 1) were also collected in aspirations.

### Blood meal analysis

Blood meal analysis data from the Gadsden-1 site from June 2015 through August 2017 indicated that both *C. stellifer* and *C. venustus* fed upon EHDV hosts throughout the year, including during periods of active EHDV transmission. The total trapping effort resulted in the collection of 116,007 *Culicoides*, 3154 of which were blood-engorged (2.7% of total collection). Blood-engorged individuals were collected from 17 different species including 2555 blood-engorged *C. stellifer* and 72 blood-engorged *C. venustus*. Successful host matches were made for 2060 *C. stellifer* (80.6% of specimens) resulting in 848 white-tailed deer, 307 fallow deer and 507 *Cervus* spp. blood meals (Fig. 2b). Blood meal analysis was successfully performed on 63 *C. venustus* (87.5% of specimens) resulting in 35 white-tailed deer, 4 fallow deer, and 7 *Cervus* host matches (Fig. 2c). The remaining 398 *C. stellifer* and 17 *C. venustus* blood meals came from other hosts not known to be hosts of EHDV. These data indicate that both of these putative vector species feed on all three of these host species, with feeding occurring throughout the late summer and fall, established transmission periods for EHDV [39].

### Seasonal abundance of *C. stellifer* and *C. venustus*

**Fig. 3:**
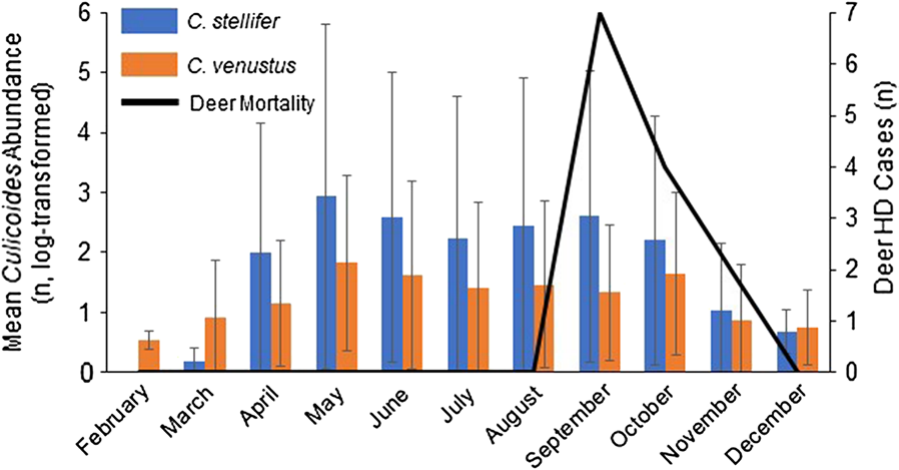
Seasonality of *C. stellifer*, *C. venustus*, and EHDV related mortality at the Gadsden-1 farm in 2016. Collections were made using CDC miniature light traps with black light LED arrays set throughout the Gadsden-1 property from January-December (20 traps used April-October, 10 traps November-March), 2016. Mean abundance and standard deviation of midges collected per night during each month are shown. Data have been log-transformed for clarity

In 2016, collections were made two nights per week on the Gadsden-1 property with 20 traps from April-October and ten traps from January-March and November-December. The first collections for both *C. stellifer* and *C. venustus* were made in March and continued through December. Average nightly abundance of *C. stellifer* was greater than 100 individuals from April through October with highest average abundance occurring in May $\left( {\bar{x} = 860,\sigma = 7 6 2. 2 2} \right)$. The highest average abundance for *C. venustus* was also observed in May $\left( {\bar{x} = 68,\sigma = 2 9.0 7} \right)$ with abundance fluctuating between 21–43 average individuals per night from June through October. The greatest period of EHDV-associated white-tailed deer mortality was observed in September with 7 deaths occurring during that month, during which on average 402 *C. stellifer* (σ = 121.42) and 21 *C. venustus* (σ = 13.67) females were collected per night of trapping (Fig. 3).

### Virus detection in field-collected midges from Alabama

In a non-outbreak setting, biting midges were sampled in 2016 in a county in Alabama where EHDV was suspected to be enzootic. Sampling effort spanned mid-summer into early fall, a time of year when HD outbreaks are most likely to occur in the southeast [35]. Twelve *Culicoides* species and 6815 midges were collected in total during the sampling period across nine trapping sites. Collections were dominated by *C. stellifer* (*n* = 4775), which outnumbered the second most abundant species, *C. arboricola*, by a ratio of greater than 6:1 (Table 4). For most species, temporal patterns of abundance were bimodal, with peaks occurring in late June/early July and early September. All species sampled, except for *Culicoides nanus* Root & Hoffman and *Culicoides crepuscularis* Malloch, were at relatively high abundance when sampling began but were collected in lower numbers from late-July through August. In early September, all species with substantive representation in the dataset, including *C. stellifer* and *C. venustus*, reached a second peak in abundance. Given that September is the high point of the EHDV-transmission season in the region [35], it is noteworthy that 57.2% of all *C. stellifer* and 39.4% of all *C. venustus* sampled were collected during September. Except for *Culicoides arboricola* Root & Hoffman and *Culicoides haematopotus* Malloch, midge numbers decreased dramatically after mid-September and only a few individuals of any species were collected by the end of the sampling period in November.

**Table 4:** Data on *Culicoides* sampled during a non-epizootic period in Alabama from June-November, 2016

Species	*Culicoides* sampled	EHDV(+) pools/Pools screened	Mean pool size
*C. arboricola*	722	0/16	5.5
*C. crepuscularis*	3	0/1	1
*C. debilipalpis*	88	0/5	2.2
*C. guttipennis*	28	0/2	1
*C. haematopotus*	512	0/17	4.9
*C. hinmani*	23	0/2	2
*C. nanus*	18	0/1	1
*C. obsoletus/sanguisuga*	78	0/1	3
*C. paraensis*	132	0/5	1.2
*C. stellifer*	4,775	0/42	11.6
*C. venustus*	99	1/5	3
*C. villosipennis*	337	0/7	4.3
Total	6815	1/104	3.4

*Notes*: *Culicoides* were collected using CDC miniature light traps at the Piedmont Research Unit of the Alabama Agricultural Experiment Station. Note that only a subset of the midges sampled were screened for EHDV

A total of 731 *Culicoides* collected from the Alabama location were screened for presence of EHDV vRNA. Twelve species of biting midge were screened in 104 pools with an average of 3.4 females per pool (Table 4). A single EHDV-positive sample was detected from a pool of *C. venustus* out of five total pools screened for this species and 15 females in all. No other biting midges were positive for EHDV RNA by qRT-PCR. The greatest number of midges screened were *C. stellifer* (486 females), followed by *C. arboricola* (88 females) and *C. haematopotus* (84 females), all of which were negative. Other biting midges screened (all negative) included *C. crepuscularis* (*n* = 1), *C. debilipalpis* (*n* = 11), *Culicoides guttipennis* (Coquillett) (*n* = 2), *Culicoides hinmani* Khalaf (*n* = 4), *C. nanus* (*n* = 1), *C. obsoletus* (*n* = 3), *C. paraensis* (*n* = 6), and *Culicoides villosipennis* Root & Hoffman (*n* = 30).

## Discussion

The identification of 20 EHDV-positive *Culicoides* pools at Florida farms where active transmission of EHDV resulting in clinical disease was occurring supported the incrimination of two probable vector species in northern Florida: *C. stellifer* and *C. venustus*. *Culicoides stellifer* has been implicated by other studies due to its great abundance during outbreaks [21–23]; however, *C. venustus* has not received much attention as a potential EHDV vector [22]. Despite this, *C. venustus* accounted for the majority of EHDV-positive pools and had higher infection rates than *C. stellifer* at all farms in this study. *Culicoides venustus* was also found to be positive for EHDV in Alabama, despite overall greater abundance of *C. stellifer* and four other species. *Culicoides venustus*, along with *C. insignis*, is a member of subgenus *Hoffmania*, a mostly tropical subgenus with several vector species [40].

Our finding that serotypes were largely equivalent between midge pools and animal samples, except for the Jefferson county farm, provides additional evidence for implicating these species as vectors of EHDV in the southeastern USA. For the Jefferson county farm, the result that EHDV-2 was only identified in *Culicoides* pools and not recovered from deer samples could be attributable to a variety of causes. Wild deer populations surrounding this farm may have had a greater prevalence of EHDV-2 than farmed populations leading to the dissimilarities in serotype distribution between deer and midges on this property. *Culicoides* are also believed to travel on wind currents [41, 42], which could have transported midges from an area experiencing greater EHDV-2 activity towards the Jefferson County farm. Finally, this result could also be attributable to stochasticity due to low sample sizes of EHDV positive animals available for sampling at the time of death.

Models indicated that abundance of both *C. stellifer* and *C. venustus* as well as the presence of CO_2_ were the most important factors in predicting EHDV positives, which has implications for future EHDV detection studies. The use of CO_2_ led to increased collections of midges (10,951 individuals were collected with CO_2_ while only 5963 were collected without CO_2_, although CO_2_ use was not recorded for 2086 individuals), which is a trend seen in other *Culicoides* studies investigating the utility of CO_2_ use with light traps [43, 44]. Future studies investigating *Culicoides* vectors should prioritize using CO_2_ to increase the likelihood of collecting sufficient sample sizes for EHDV detection. The inclusion of *C. stellifer* and *C. venustus* abundance in this model further reinforces their vector capacity. Despite the “other species” category having many more individuals than the *C. stellifer* or *C. venustus* fields on several trapping dates, it is still the abundance of these two species specifically that predicts EHDV positives in samples.

While both *C. stellifer* and *C. venustus* were included in the most parsimonious model, our data indicate that abundance of *C. venustus* collected was the only factor between models that significantly affected EHDV outcome with greater abundance of *C. venustus* resulting in an increased probability of detecting positive pools. This speaks to the likely greater significance of this species as a vector of EHDV in these areas. Additionally, the higher total infection rates for this species at each site lends strength to this assertion. *Culicoides venustus* vector competence for both BTV and EHDV has been tested in one population from New York. One positive infection each for BTV and EHDV-1 out of 141 and 38 midges, respectively, orally exposed to virus was identified in this species; however, transmission was not demonstrated [45]. The authors determined that *C. venustus* is likely not an efficient vector in New York. However, evidence from other vector-borne pathogen systems indicates that different populations of the same species can have variable vector competence [46, 47]. Southeastern *C. venustus* populations may be more susceptible to EHDV infection than the New York populations evaluated previously [45]. Further, the dissemination rate and transmission potential of *C. venustus* has not been determined with any populations of this species. While greater infection rates in *C. venustus* may indicate greater competence for the virus by this species, it is possible that the far greater abundance of *C. stellifer* on the landscape may overcome any dearth in competence [48].

Our finding that light type is not a significant factor in collecting EHDV positive pools of *Culicoides* has implications for the effect of orbivirus infection on light perception in *Culicoides*. A recent study indicated that BTV infection of *C. sonorensis* leads to an aversion for UV light [49]. EHDV-positive pools were collected using both UV and incandescent light in the present study indicating that at least some EHDV-infected midges were attracted to UV light. However, it is possible that light aversion could be seen in some individuals resulting in a lower calculated infection rate that does not adequately represent the actual infection rate on these properties. It is also possible that the UV light aversion is limited to BTV infection or is not uniform across *Culicoides* species. Additional research into this topic should be pursued to better understand how both of these orbiviruses may affect *Culicoides* physiology.

Previous studies at the Gadsden-1 site provided evidence for the frequent use of EHDV hosts for blood meals by *C. stellifer* and *C. venustus*. These data fulfill the criterion that an interaction in time and space between hosts and incriminated vectors be demonstrated. Blood meal analysis data revealed that both species not only feed on white-tailed deer in great abundance but also feed on fallow deer and elk, species that are competent at maintaining a viremia to EHDV [37, 38]. Furthermore, a pattern of preference for fallow deer and elk by *C. stellifer* on big game preserves has been found [36]. Currently, the roles and significance of these two ruminant species in the transmission and maintenance of EHDV are as yet unknown on big game preserves in Florida and may warrant additional studies.

While the present study aimed to determine the vectors in the southeastern USA, the range of both *C. stellifer* and *C. venustus* could have implications for their vector status in other regions as well. Both species are known to occur north to Canada, with *C. stellifer* occurring throughout the USA except the far northwestern states of Washington and Oregon and *C. venustus* primarily occurring east of Nebraska [50]. Due to the high infection rates in this study and the low abundance of *C. sonorensis* in the eastern USA, *C. venustus* may be acting as the dominant vector of EHDV throughout a large portion of this region. The lower infection rates seen for *C. stellifer* and the extensive range of this species could indicate that this species is acting as a secondary vector for EHDV throughout the USA. Virus detection studies should be pursued throughout the range of both species to better understand their role as vectors of EHDV in the USA.

Despite testing 17 additional species, including species that are abundant on the landscape and have been implicated previously, no additional species were found positive for EHDV viral RNA. *Culicoides debilipalpis* has been implicated multiple times in other studies [21, 51] and was present in our collections from all five farms sampled, but no pools of *C. debilipalpis* tested positive for virus. Overall populations of *C. debilipalpis* were low during the 2017 EHDV outbreak, and only 28 pools were tested. Due to this low abundance and lack of virus positives, we do not believe that this species is a primary vector of EHDV in this region. *Culicoides insignis*, a confirmed vector of BTV [52] also present at all five deer farms, was the second most abundant *Culicoides* species collected, and constituted the majority of pools from Liberty County. Despite this, no samples from this species were positive for EHDV viral RNA. A further 46 pools of *C. haematopotus*, the third most abundant species collected, also tested negative for EHDV viral RNA. Due to the lack of virus positives despite exhibiting great relative abundance, it is unlikely that either *C. insignis* or *C. haematopotus* are vectors of EHDV in this region. This lack of positives from abundant species, combined with our finding of multiple positives from both *C. stellifer* and *C. venustus*, also indicate that the virus positive pools detected were likely not false positives or contaminated samples and represented true infections in those *Culicoides* species.

Unfortunately, despite the large-scale virus testing conducted, only one pool of *C. stellifer* tested positive for the presence of BTV viral RNA at the Gadsden-2 site. Interestingly, despite testing over 5000 *C. insignis*, no BTV positive samples were detected from this confirmed BTV vector species [52]. Previous virus detection studies in Louisiana and Florida failed to detect BTV in 884 and 200 individual *C. stellifer,* respectively [53, 54]. While intrathoracic inoculations of BTV into *C. stellifer* have resulted in low infection rates, infection through viral blood meals has not been successfully demonstrated for this species [26]. *Culicoides stellifer* may be involved in BTV transmission; however, based on the low infection rate in the present and other studies, it is unlikely that *C. stellifer* is playing a dominant role in the transmission of this pathogen.

There were some limitations to this study. The first was the lack of variation in trap heights. Many *Culicoides* species in the Florida panhandle, including *C. stellifer*, *C. debilipalpis*, *C. haematopotus* and *C. venustus*, are known to frequent forest canopies, often descending to take blood meals and then moving back into the forest canopy [35]. Our collection of midges at one height may have led to underestimations of virus-positive midges in this study. Another limitation is our inability to draw inferences about EHDV transmission on the natural landscape. We do not have adequate data on EHDV prevalence or serotypes found in wild populations to determine whether the patterns observed at the deer farms are reflected in the natural ecosystems of north Florida.

## Conclusions

The present study has identified two species, *C. stellifer* and *C. venustus*, as probable vectors of EHDV in the southeastern USA, fulfilling two of the four World Health Organization criteria for vector recognition for both of these *Culicoides* species. Viral RNA was detected in field-collected individuals of both species lacking blood in the gut and an interaction in time and space between the host, white-tailed deer, and the putative vector species was demonstrated. While the last two criteria, showing infection and transmission potential for Florida populations of these two species, have not been fulfilled yet, we believe it is of the utmost importance to establish the most likely vector species of EHDV in this region. Identification of the vector species can lead to more targeted control efforts for deer farmers in the state and direct future studies on *Culicoides* ecology and biology towards those species most likely to transmit pathogens.

## Data Availability

The complete dataset of species pools tested for virus and corresponding Cq values are available upon request.

## Abbreviations

BTV: Bluetongue virus
cDNA: complementary DNA
EHDV: epizootic hemorrhagic disease virus
HD: hemorrhagic disease
MLE: maximum likelihood estimate
qRT-PCR: quantitative reverse transcription polymerase chain reaction
vRNA: viral RNA
